# Correction to: Elobixibat Effectively Relieves Chronic Constipation in Patients with Cancer Regardless of the Amount of Food Intake

**DOI:** 10.1093/oncolo/oyad089

**Published:** 2023-03-25

This is a correction notice for: Anna Ozaki, Takaomi Kessoku, Yuki Kasai, Yuma Takeda, Naoki Okubo, Michihiro Iwaki, Takashi Kobayashi, Tsutomu Yoshihara, Yasushi Honda, Akiko Fuyuki, Takuma Higurashi, Hiroto Ishiki, Masataka Taguri, Shunsuke Oyamada, Noritoshi Kobayashi, Atsushi Nakajima, Yasushi Ichikawa, Elobixibat Effectively Relieves Chronic Constipation in Patients with Cancer Regardless of the Amount of Food Intake, *The Oncologist*, Volume 26, Issue 10, October 2021, Pages e1862–e1869, https://doi.org/10.1002/onco.13879

In the originally published version of this article, the Disclosures section was incorrect and should have read as follows:

This study was conducted without company funding; however, three co-authors have financial relationships to disclose. Takaomi Kessoku received lecture honoraria and research funding from EA Pharma Co. Ltd. and Mochida Pharmaceutical Co., Hiroto Ishiki received lecture honoraria from EA Pharma Co. Ltd., and Atsushi Nakajima received scholarship fee, lecture honoraria, and research funding from EA Pharma Co. Ltd. and Mochida Pharmaceutical Co.

These details have been amended only in this correction notice to preserve the published version of record.

